# Pyogenic Granuloma Mimicking T1 Colorectal Carcinoma

**DOI:** 10.7759/cureus.17536

**Published:** 2021-08-29

**Authors:** Naoki Asayama, Yukari Takeuchi, Kenjiro Shigita, Mayumi Kaneko, Shinji Nagata

**Affiliations:** 1 Department of Gastroenterology, Hiroshima City Asa Citizens Hospital, Hiroshima, JPN; 2 Department of Anatomical Pathology, Hiroshima City Asa Citizens Hospital, Hiroshima, JPN

**Keywords:** magnifying endoscopy, t1 (deep submucosal invasive) colorectal carcinoma, total excisional biopsy, endoscopic mucosal resection, pyogenic granuloma

## Abstract

Pyogenic granuloma (PG), a benign capillary hemangioma, is extremely rare in the colon. Here, we present a case of PG that was difficult to distinguish from T1 (deep submucosal invasive) colorectal carcinoma. A 57-year-old woman with no remarkable history was referred to us for a detailed investigation of a positive fecal occult blood test. Colonoscopy revealed a reddish, irregular-shaped, protruding lesion (5 mm) in the rectum. We performed endoscopic mucosal resection of the lesion as total excisional biopsy because T1 colorectal carcinoma was suspected, despite the lesion’s small size after observation by magnifying endoscopy. Histologically, the protruding lesion mainly consisted of numerous capillaries lined with plump and flat endothelial cells without signs of malignancy. Colorectal carcinoma, on the other hand, is composed of tall columnar atypical epithelial cells showing neoplastic proliferation. Thus, cell morphology is completely different between PG and colorectal carcinoma. The final diagnosis was colonic PG with a negative vertical margin. In conclusion, physicians should be aware of a colorectal protruding lesion devoid of malignant potential, as in this case, where the lesion was difficult to diagnose accurately and to distinguish from T1 colorectal carcinoma on magnifying endoscopy. Physicians should consider PG as a differential diagnosis in similar cases.

## Introduction

Pyogenic granuloma (PG) is a benign capillary hemangioma that forms granulomas due to secondary inflammation. PG develops in various age groups from infants to the elderly [1-3]. It commonly occurs on the skin, lips, gums, and tongue. Although there have been some reports of PG in the gastrointestinal tract, particularly the esophagus and small intestine, it is extremely rare in the colon [1-3]. Here, we present a case of PG that was difficult to distinguish from T1 (deep submucosal invasive) colorectal carcinoma.

## Case presentation

**Figure 1:**
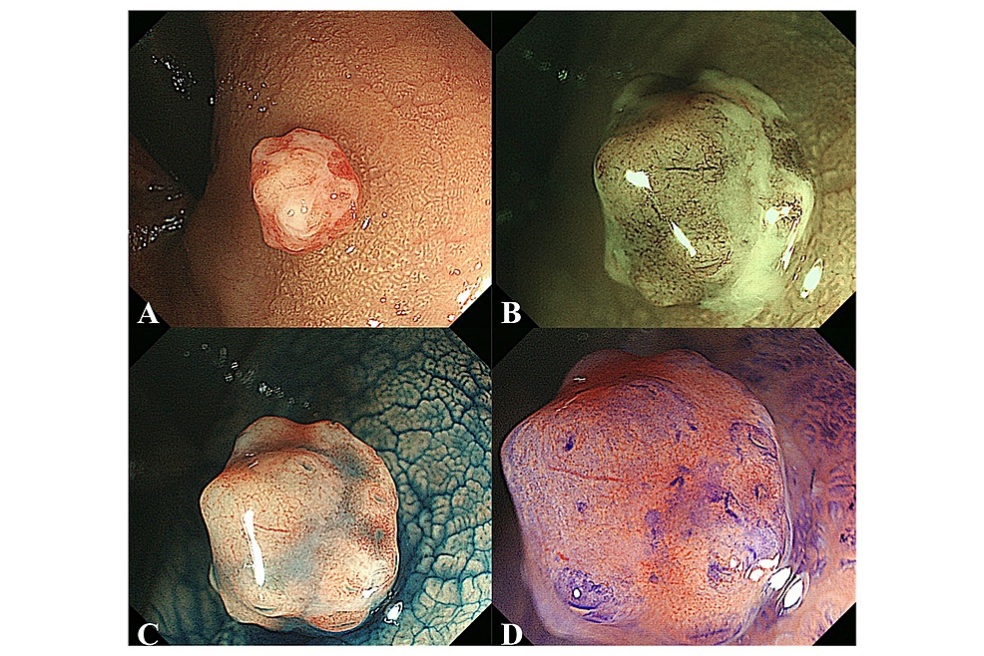
Findings on colonoscopy. A: Colonoscopy revealed a reddish, irregular-shaped, protruding lesion covered with white exudate in the rectum. B: Magnifying endoscopy with narrow-band imaging revealed that the vessel pattern had loose vessel areas and interruption of thick vessels, and the surface pattern had amorphous areas. C: After spraying with indigo carmine dye, the lesion and its margins became more clearly visible. Magnifying endoscopy with indigo carmine spraying revealed a smooth surface without mucosal pits. D: Magnifying colonoscopy with crystal violet staining revealed a type VN pit pattern.

A 57-year-old woman with no remarkable history was referred to us for a detailed investigation of a positive fecal occult blood test. She had no family history of colorectal carcinoma. Physical examination was unremarkable, and laboratory tests showed no abnormalities. Colonoscopy revealed a reddish, irregular-shaped, protruding lesion (5 mm) in the rectum covered with white exudate. The lesion was diagnosed as an epithelial tumor because its coloration, surface structure, and base were different from the surrounding colorectal mucosa and the boundary between the lesion and the surrounding mucosa was clear. This diminutive polyp had neither fold convergence nor expanding appearance on nonmagnified endoscopic images (Figure 1A). Magnifying endoscopy with narrow-band imaging (NBI) revealed that the vessel pattern had loose vessel areas and interruption of thick vessels, and the surface pattern had amorphous areas (Figure 1B). Based on the NBI findings, the lesion was diagnosed as type 3 according to the Japan NBI Expert Team classification [4]. Chromoendoscopy with indigo carmine dye spraying clearly showed the lesion’s margin and a shallow depressed area in the center of the tumor. Magnifying endoscopy with indigo carmine dye spraying revealed a smooth surface without mucosal pits (Figure 1C). Magnifying endoscopy with crystal violet staining revealed a type VN (N: nonstructural) pit pattern [5] (Figure 1D).

**Figure 2:**
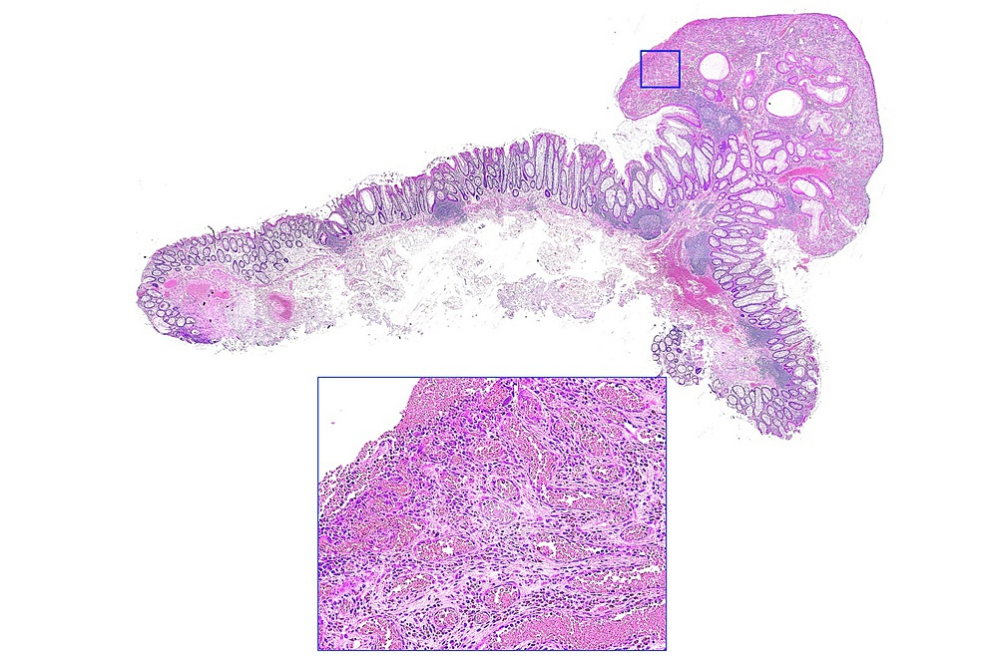
Findings of pathologic examination. Histology confirmed the lesion to be pyogenic granuloma with a lobular proliferation of variably sized capillaries in edematous stroma without any signs of malignancy.

Because deep submucosal invasive colorectal carcinoma was suspected despite the lesion’s small size, total excisional biopsy was performed by endoscopic mucosal resection (EMR). Histologically, the protruding lesion had some erosions on the surface and consisted of mainly neoplastic hyperplasia of capillaries without any signs of malignancy. A high-power view of the polypoid lesion showed dilatation and proliferation of capillaries and edematous stroma containing inflammatory cell infiltrates (Figure 2). The final diagnosis was colonic PG with a negative vertical margin. Surveillance colonoscopy will be performed at one year of follow-up.

## Discussion

PG is a benign lesion whose etiology is still under discussion [1-3]. It develops as a result of trauma or injury or is associated with stroma undergoing secondary inflammation and granulation tissue reaction. Most PG lesions in the gastrointestinal tract are incidental findings during colonoscopy for unrelated reasons. PG of the colon is often found following anemia, positive fecal occult blood test, or overt bleeding because the lesion consists of hyperplasia and dilated capillaries and often has erosions and ulcers on the surface [1-3].

Macroscopically, PG is usually described as a red or dark red polypoid lesion (pedunculated or semi-pedunculated) with surface ulceration or a white fur that bleeds easily upon contact. Therefore, PG can be misdiagnosed as colorectal carcinoma, highlighting the importance of histology for a definitive diagnosis. Microscopically, PG is best described as a capillary hemangioma surrounded by inflammatory cells in a loose connective tissue stroma. PG comprises numerous capillaries lined with plump and flat endothelial cells, with an edematous stroma containing acute and chronic inflammatory cell infiltrates. Colorectal carcinoma, on the other hand, is composed of tall columnar atypical epithelial cells showing neoplastic proliferation. Thus, cell morphology is completely different between PG and colorectal carcinoma.

Non-neoplastic lesions are generally white or of the same color as the surrounding colorectal mucosa as observed on a regular colonoscopic view. In this case, the diminutive polyp was reddish and irregularly shaped, and had neither fold convergence nor expanding appearance on nonmagnified endoscopic images. We diagnosed this lesion as a malignant tumor based on these endoscopic findings from a regular endoscopic view. A shallow depressed area in the center of the tumor was seen on chromoendoscopy with indigo carmine dye spraying. Furthermore, the tumor was not completely covered with exudate, enabling us to wash away part of the exudate and observe the surface in detail by magnifying endoscopy. However, we misdiagnosed the lesion as T1 colorectal carcinoma based on the findings. Both fold convergence and expanding appearance have been reported as characteristic findings of T1 carcinoma observed from a regular endoscopic view, but these findings are difficult to recognize for diminutive tumors. On the other hand, diminutive tumors with depressions have been reported to have a high frequency of carcinoma and submucosal invasion [6]. Therefore, the lesion, in this case, was difficult to distinguish from T1 colorectal carcinoma because the depressed part of the polypoid lesion had nonstructural pits (type VN pit pattern) with crystal violet staining. There has been no similar report of colonic PG mimicking T1 colorectal carcinoma on magnifying endoscopy with NBI, indigo carmine dye spraying, and crystal violet staining.

Magnifying endoscopy findings corresponded to the description of the capillaries in the pathologic findings. We speculate that the overreactive growth of inflammatory granulation tissue after irritation might have caused this polypoid lesion and that variously sized capillaries and stromal inflammation proliferated and increased in density. The vessel pattern and surface pattern were difficult to observe because the passage of light for NBI was impeded by the growth and dilatation of small capillaries in the lamina propria. The lesion needed to be removed by EMR for accurate diagnosis.

PG is devoid of malignant potential but may recur even after apparently complete resection [7]. Therefore, surveillance colonoscopy is necessary.

## Conclusions

We reported a rare case of PG of the colon that required differentiation from T1 carcinoma. We did not diagnose PG from the endoscopic findings and performed EMR as total excisional biopsy based on a diagnosis of T1 carcinoma, resulting in a definitive diagnosis. Physicians should be aware of a colorectal protruding lesion devoid of malignant potential, as in this case, where the lesion was difficult to diagnose accurately and to distinguish from T1 colorectal carcinoma on magnifying endoscopy.
